# Impact of social determinants of health on obesity among American Indian and Alaska Native young adults

**DOI:** 10.1371/journal.pone.0322164

**Published:** 2025-05-02

**Authors:** Kimberly R. Huyser, Angela G. Brega, Margaret Reid, Tassy Parker, John F. Steiner, Jenny Chang, Luohua Jiang, Amber L. Fyfe-Johnson, Michelle Johnson-Jennings, Vanessa Y. Hiratsuka, Nathania Tsosie, Spero M. Manson, Joan O’Connell

**Affiliations:** 1 Department of Sociology, University of British Columbia, Vancouver, BC, Canada; 2 Centers for American Indian and Alaska Native Health, Colorado School of Public Health, University of Colorado Anschutz Medical Campus, Aurora, Colorado, United States of America; 3 Department of Health Systems, Management, and Policy, Colorado School of Public Health, University of Colorado, Denver, Colorado, United States of America; 4 Department of Family and Community Medicine, University of New Mexico School of Medicine, Albuquerque, New Mexico, United States of America; 5 Institute for Health Research, Kaiser Permanente Colorado, Aurora, Colorado, United States of America; 6 Department of Medicine, University of Colorado Anschutz Medical Campus, Aurora, Colorado, United States of America; 7 Department of Medicine, School of Medicine, University of California Irvine, Irvine, California, United States of America; 8 Department of Epidemiology, School of Public Health, University of California Irvine, Irvine, California, United States of America; 9 Institute for Research and Education to Advance Community Health (IREACH), Department of Medical Education and Clinical Sciences, Washington State University, Seattle, Washington, United States of America; 10 Indigenous Wellness Research Institute, University of Washington, Seattle, Washington, United States of America; 11 Southcentral Foundation, Research Department, Anchorage, Alaska, United States of America; 12 Albuquerque Area Southwest Tribal Epidemiology Center, Albuquerque, New Mexico, United States of America; University of Pennsylvania Perelman School of Medicine, UNITED STATES OF AMERICA

## Abstract

We examined the prevalence of obesity among American Indian and Alaska Native (AIAN) young adults and to investigate the association between key social determinants of health (SDOH) and higher body mass index (BMI). We used the Indian Health Service Improving Delivery Data Project from fiscal year 2013. It includes data for 20,698 AIAN young adults aged 18–24 years. We added county-level measures of SDOH from the USDA Food Environment Atlas and the Census as contextual variables. We conducted stratified logistic regressions to understand the relationship between these SDOH indicators and odds of obesity. Thirty-seven percent of our sample was identified as obese (i.e., BMI ≥30). Individuals who lived in counties with lower levels of educational attainment and higher levels of poverty had higher odds of obesity than those who lived in counties with higher education and lower poverty (p < 0.0001). Counties with higher poverty rates had less access to social and environmental resources than the lower poverty rate counties (p < 0.0001). Federal and state governments should increase access to education and economic development opportunities to positively impact health outcomes.

## Introduction

Obesity is a known risk factor for type 2 diabetes (T2DM), cardiovascular disease, cancer, and premature mortality [1]. In the US, American Indian and Alaska Native (AIAN) peoples have the highest prevalence of diabetes, twice that of the general population [2]. Among AIAN adults age 18–44, T2DM prevalence has decreased significantly from 2013 to 2017, T2DM remains a major health concern for AIAN, with AIANs having the highest rates of T2DM compared to all other racial/ethnic groups in the USA [3]. In 2018, AIAN youth had the second highest prevalence of T2DM [4], and AIANs over 20 had a higher prevalence of T2DM than all other races and ethnicities [2].

AIAN persons who are 18 years old and older are significantly more likely to be obese than their non-Hispanic White (NHW) counterparts [5]. Unfortunately, obesity is also associated with increased likelihood of experiencing discrimination – institutionally, interpersonally, and in the labor market [6]. AIAN childhood obesity remains higher than the US obesity rate for children of all racial groups [7]. Given the association with obesity and T2DM along with other chronic diseases and social discrimination, it is important to consider the factors that influence body weight. Our study extends the understanding of obesity among AIAN young adults from ages 18–24 years old. It examines the association of the social environment and conditions on the risk of obesity for young AIAN adults.

**Fig 1:**
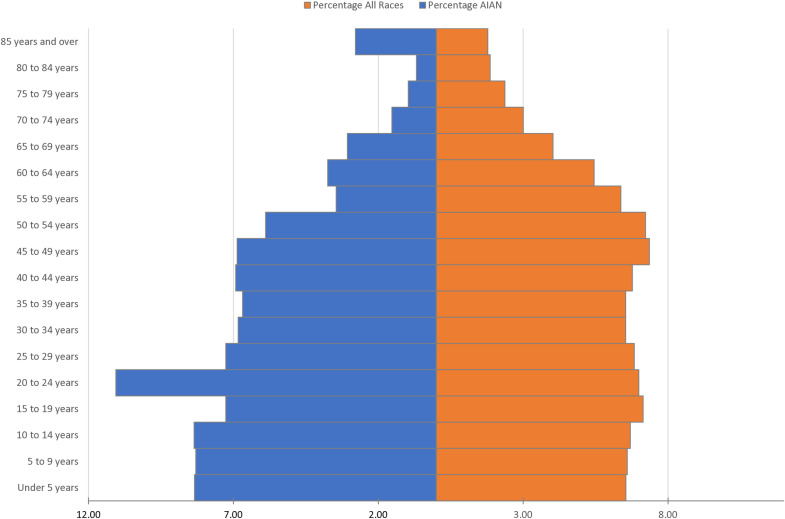
Population pyramid for AIAN peoples alone and all races in the United States. Visual illustration of percentage of AIAN populations compared to percentage of all race by age cohort. Source is census 2010 SF-1.

Since the accumulation of health risks in early life can later lead to increased morbidity/mortality and life chances, it is important to identify key periods across the life course during which individuals may positively impact their outcomes [8]. Emerging adulthood is the period between ages 18 to 25 in which an individual transitions from adolescence to adulthood [9]. During this period, individuals become responsible for self-management of their health and develop health-related behaviors and habits that will impact future health outcomes [10]. Furthermore, AIAN youth have a higher prevalence of obesity than their NHW counterparts [10]. Fig 1 presents a population pyramid of the US population and the AIAN population; the AIAN population is younger (less than 35 years old) than the overall US population (see Fig 1). Given the larger proportion of young adults among AIAN populations, it is useful contribution to the literature to examine a portion of the AIAN populations that is often overlooked in lieu of children and older adults [10–14].

Young adulthood is one of the time periods in an individual’s life that can influence life course outcomes [6,9], the social environment and conditions in which the individual is born, grows, lives, and works also influences health status and health outcomes, and these factors are the social determinants of health (SDOH) [15,16]. Extant literature has demonstrated that at the individual level, AIAN adults have higher rates of poverty, lower levels of educational attainment, and have lower rates of health insurance coverage, but these studies lack a health status dependent variable [17–19]. In this study, we use data from the Indian Health Services (IHS), an agency within the US Department of Health and Human Services, which lacks individual level socioeconomic status. Thus, we use county-level data from the Census Bureau and United States Department of Agriculture to provide the socioeconomic and SDOH context for our AIAN sample. We use the Healthy People 2020 SDOH framework to organize our independent variables, and we have four major domains (education & economic stability, healthcare, neighborhood & built environment, and social & community context) [16].

The current study examined the risk of obesity among AIAN young adults (ages 18–24). It also examined the association between key SDOH indicators and body mass index (BMI) and used the STROBE approach to describe our study and findings [20]. One of the benefits of the study was that we drew upon data from the Indian Health Services (IHS), which is an agency within the US Department of Health and Human Services and serves serve 2.6 million AIAN people. IHS is the principal federal healthcare provider and health advocate for 574 federally recognized Tribes across the US [21]. The combination of IHS data with Census Bureau and United States Department of Agriculture allows the study to examine the link between SDOH county level indicators and the risk of obesity among AIAN young adults.

## Materials and methods

### Data source

Data for this analysis were extracted from the IHS Improving Health Care Delivery Data Project (henceforth the IHS Data Project). The IHS Data Project is comprised from administrative IHS electronic data: the National Data Warehouse (NDW) and Purchased and Referred Care (PRC) data [22]. The IHS Data Project was a purposeful sample of AIAN persons who lived in any one of 15 IHS Service Units (hereafter referred to as project sites) located throughout the United States, including one project site in the East, four in the Northern Plains, two in the Southern Plains, five in the Southwest, two on the Pacific Coast, and one in Alaska. These 15 project sites include 72 counties. These sites were consistent with geographic regions used in other studies [10]. The IHS Data Project population is comparable to the national IHS service population in terms of age and gender [23]. IHS healthcare resources are severely strained. IHS 2019 per capita expenditures were limited to $4,078, in stark contrast to $9,726 for the US population in 2017 as a whole [24].

The IHS Data Project did not include individual level SDOH indicators. Thus, SDOH county-level measures were added to the IHS Data Project by county and were drawn from Census Bureau and United States Department of Agriculture (USDA) Food Environment Atlas data sources. From the Census, we included county-level indicators from the 2010–2014 American Community Survey (ACS) and decennial Census 2000 and 2010.

#### Approvals, guidance, and oversight.

Project personnel collaborate with IHS and the Tribal organizations that participate in IHS Data Project. This collaboration takes place through the project’s Collaborative Network, which includes three advisory committees (i.e., Steering, Project Site, and Patient) and we obtained written approvals from IHS National Institutional Review Board (IRB), Tribal IRBs, Tribal Councils, or Tribal Authorities, in addition to the collaborating university’s IRB, the Colorado Multiple Institutional Review Board. Specific approving Tribal entities are not named to protect community confidentiality, as requested by participating sites.

#### HIPAA waiver of documentation of informed consent and waiver of HIPAA authorization.

The data analysis is a secondary data analysis. The Indian Health Service has approved a Waiver of Documentation of Informed Consent In accordance with 45 CFR 46.116(d) and 45 CFR 46.117(c), and a Waiver of HIPAA Authorization in accordance with 45 CFR § 164.512(i).

### Study sample

The data set includes data for 20,698 AIAN young adults aged 18–24 years who were IHS active users during fiscal year (FY) 2013, July 1, 2012 to June 30, 2013, and had an age-appropriate BMI value within biologically plausible ranges. An FY2013 IHS active user was defined as a patient who obtained services at least once during the current or the preceding 2 FYs (FY2011-2013 for FY2013 active users). Exclusion criteria included 1) pregnancy anytime during FY2013 (n = 526); 2) malignant cancer, kidney disease, or treatment for a transplant or an amputation (n = 95); 3) missing data for community- and county-level measures of SDOH (n = 452); or 4) enrolled in Medicare in FY2013 (n = 69). After these exclusions, the sample included 20,698 young adult active users with a valid measure of BMI.

### Measures

#### Obesity.

Our primary outcome variable was obesity (obese: BMI ≥30.0 kg/m^2^) using the standard definitions for obesity for individuals age 18 and older. We excluded biologically implausible height, weight, and BMI values for adults aged 18 years and older in several steps based on similar work reported elsewhere [25]. We matched remaining height and weight values by date of service and calculated BMI as weight and kilograms divided by height in meters squared.

#### Demographic variables.

The NDW provides data on age and sex. Service dates and month and year of birth were used to calculate the age for each BMI measurement.

#### SDOH indicators.

**Fig 2:**
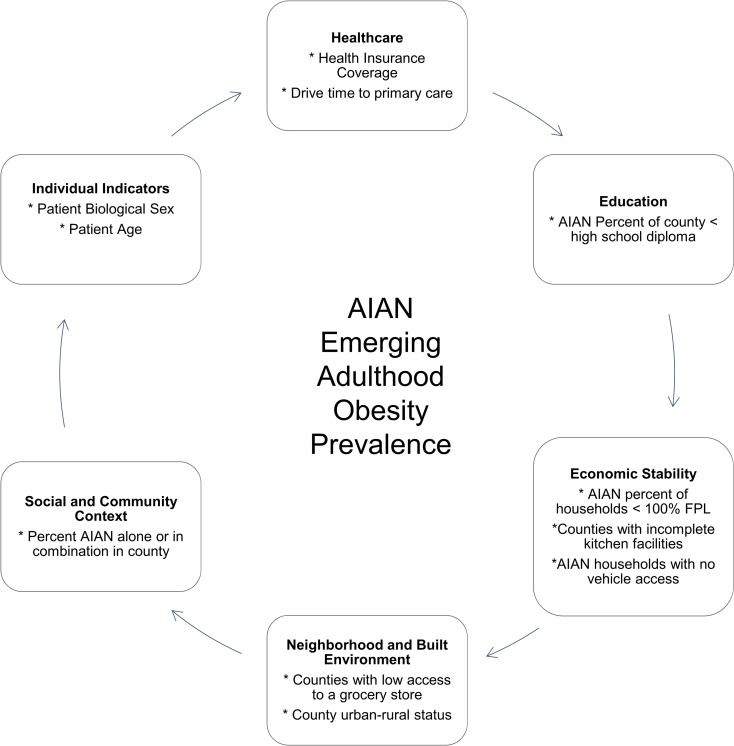
Study SDOH conceptual framework. SDOH conceptual framework drawn from Health People 2020 and factors potentially influencing AIAN young adulthood obesity prevalence.

Fig 2 illustrates how we grouped our variables within the Health People 2020 SDOH Framework [16]. We used education and income measures from a 2010–2014 ACS county-level data special tabulation by the U.S. Census Bureau in which they calculated estimates for people who self-reported being AIAN alone or AIAN in combination with other races, and who reported access to IHS services in the ACS [26]. Educational attainment was defined as the percentage of adults aged 25 years and older who did not complete high school; the median county level value for this educational attainment measure was 46.0%. We defined the percentage of households with a low income as the percentage with incomes below 100% of the federal poverty level (FPL). Across the counties in this study, the median percentage of households under 100% of the federal poverty level was 27.9%. From the decennial Census 2000, we used the percentage of single-race AIAN households with incomplete kitchen facilities (median was 1.8%) and no vehicle access by county (median was 12.9%), which was downloaded from U.S. Census publicly available tabulations [27,28]. The IHS Data Project includes person-level health insurance coverage (categories: Medicaid, Private, or None) and links respondents to the IHS site most often used*.* Measures of access to medical care included county rurality, county categorization, and drive times to the nearest IHS facility that provided primary care services (i.e., drive time categories: <30 minutes and 30+ minutes). Drive times to the nearest clinic were estimated from a central location in each community to the clinic location and the Stata program, osrmtime [29].

From the USDA Food Environment Atlas, we used food-access measures including the percentage of the population with low access to a grocery store in 2010 [30]. The median was 25.3% across our 72 counties. We also coded county rurality using the National Center for Health Statistics urban-rural classification. From the decennial Census 2010, we included the percentage of people in each county that identify as AIAN persons alone and in combination with one or more other races. The median was 14.6% for our 72 counties.

### Statistical analysis

At the county level, we stratified the study population by poverty level. Counties were either above or below the median (27.9%) on the percentage of households living below the federal poverty level. We refer to counties below the median as lower-poverty counties and those above the median as higher-poverty counties. Descriptive statistics computed for individual demographic variables and county characteristics were examined. Prevalence of obesity was calculated for each demographic and county characteristics subgroup. Multivariable mixed logistic regression models were fitted for obesity and class III obesity with county random effects and project site fixed effects to account for clustering of observations within counties and Service Units. We used SAS® 9.4 and Stata statistical software to conduct descriptive and multivariable analyses [31,32].

## Results

Table 1 presents the characteristics of our 72 counties relative to the attributes of the aggregate level of all US counties and compares high-poverty and low-poverty counties within the study sample. We have a column for the aggregate statistic of all US counties across each of our measures. We also have one column for each set of 36 counties that are below and above the median percentage of households living below 100% FPL. Counties above the median have 27 percent or more households living below 100% FPL and are the higher poverty rate counties. Counties below the median have less than 27 percent of households living below 100% FPL and are lower poverty rate counties. The counties included in this analysis had similar percentages of people by gender/sex and age compared to the national mean percentages. Our sample counties had a higher percentage of individuals who had Medicaid and/or were uninsured than the national median. Specifically, the uninsured percentage among AIAN in all US counties was 22%, whereas it was around 30% for the counties in our sample. The percentage of AIAN without health insurance was higher in the counties with higher poverty rates than in counties with lower poverty rates (33% compared to 29.7%). Among NHWs in these counties, 14.3% and 13.8% were without health insurance in lower- and higher-poverty rate counties, respectively.

**Table 1:** Characteristics of study counties.

	All US counties	Lower poverty rate counties	Higher poverty rate counties
	N	N	N
All counties	3142	36	36
**Individual and healthcare**			
Gender^a^			
Female (mean %)	48.15%	49.05%	50.35%
Male (Mean %)	51.85%	50.95%	49.65%
Age group^a^			
18–19 years	3.15%	3.24%	3.36%
20–24 years	6.94%	7.62%	7.77%
Health insurance coverage^a^			
Medicaid All persons percentage	18.51%	16.98%	24.69%
Uninsured among AIAN persons	22.02%	29.68%	32.98%
Uninsured among 19–25 year olds	30.24%	34.31%	41.48%
**Education and economic stability**			
Educational attainment: % <high school^a^^,^^b^			
Median (AIAN in Combination & uses IHS)^c^		43.4%	48.7%
Median (All Persons over age of 25)	13.0%	11.29%	14.46%
Income^a^			
Median (All Households)	$44,798.00	$48,125.50	$41,809.50
Median (AIAN alone Households)	$36,346.00	$37,769.00	$29,488.50
Poverty status^a^^,^^d^			
Median % below 100% FPL	16.0%	14.9%	21.0%
Median % below 100% FPL (AIAN Alone)	21.3%	21.8%	41.3%
Households with incomplete kitchen facilities^e^			
Median (percent)	1.7%	2.2%	3.2%
Median (percent) (AIAN Alone Households)		1.0%	2.7%
Maximum (percent) (AIAN Alone Households)		15.4%	55.0%
Households with no vehicle access^e^			
Median (All Households)	6.78%	6.6%	8.2%
Median (AIAN alone Households)		8.1%	16.0%
**Neighborhood and built environment**			
Low access to a grocery store^f^			
Percent median	19.7%	22.6%	29.9%
NCHS 2013 Urban-rural indicator			
% of Rural	18.5%	44.4%	55.6%
**Social and community context**			
Percent AIAN alone or in combination^g^			
Median	1.00%	10.9%	21.1%

^a^Data source: American Community Survey 2010–2014 5-year estimates.

^b^Education attainment for all persons age 25 years and older.

^c^Data source: Special Tabulation American Community Survey 2010–2014 5-year estimates.

^d^Poverty status – calculated for population where determined.

^e^Data source: US Census Bureau 2000.

^f^Data source: US Department of Agriculture Food Environment Atlas.

^g^Data source: US Census Bureau 2010.

AIAN: American Indian/Alaska Native; FPL: Federal poverty level.

Table 2 presents the characteristics of our study population by level of poverty and obesity status. Sixty-two percent of our sample was female and 43.8% was 22–24 years old. Across the sample, 31.7% had Medicaid, 15% had private insurance, and 56.5% had no health insurance other than access to the IHS. Approximately 9.5% of our sample resided 30 minutes or more from a primary care facility. We saw a wider distribution between the lower-poverty counties and the higher-poverty counties within the education and economic stability variables. The higher-poverty counties also had lower levels of educational attainment than the lower-poverty counties. Further, compared to lower-poverty counties, the higher-poverty counties had a higher prevalence of households with incomplete kitchens, for instance kitchens lacking a sink with piped water, a stove, and/or a refrigerator. Similarly, in the higher-poverty counties, households were more likely to lack access to a vehicle than were households in lower-poverty counties. Likewise, there was a larger percentage of people living more than 1 mile from a supermarket or large grocery store in the higher- versus the lower-poverty counties. Compared to lower-poverty counties, a larger proportion of the higher-poverty counties were classified as rural based on the NCHS urban-rural indicator. Finally, the higher-poverty counties had a larger percentage of people who self-reported being AIAN alone or AIAN in combination with other races than did the lower-poverty counties.

**Table 2:** Characteristics by poverty level and obesity among young adults aged 18–24 years. Fiscal year 2013.

	All		Lower Poverty Rate Counties		Higher Poverty Rate Counties		p-value from Chi Square test comparing two poverty levels	BMI	
								Obese: BMI 30+	
	N	Column %	N	Column %	N	Column %		N	Row %
Total	20,698	100	9,898	47.8	10,800	52.2		7,843	37.9
**Individual and healthcare**									
Gender							0.0080		
Female	12,824	62.0	6,040	61.0	6,784	62.8		5,125	40.0
Male	7,874	38.0	3,858	39.0	4,016	37.2		2,718	34.5
Age group							0.0815		
18–19 years	5,789	28.0	2,697	27.2	3,092	28.6		1,785	30.8
20–21 years	5,842	28.2	2,832	28.6	3,010	27.9		2,194	37.6
22–24 years	9,067	43.8	4,369	44.1	4,698	43.5		3,864	42.6
Health insurance coverage									
Medicaid							<0.0001		
No Medicaid	14,140	68.3	7,336	74.1	6,804	63.0		5,289	37.4
Had Medicaid	6,558	31.7	2,562	25.9	3,996	37.0		2,554	38.9
Private insurance							<0.0001		
No private insurance	17,587	85.0	8,123	82.1	9,464	87.6		6,670	37.9
Had private insurance	3,111	15.0	1,775	17.9	1,336	12.4		1,173	37.7
Other insurance coverage							<0.0001		
No other insurance	9,001	43.5	4,031	40.7	4,970	46.0		3,480	38.7
Had other insurance	11,697	56.5	5,867	59.3	5,830	54.0		4,363	37.3
Drive time to primary care							<0.0001		
<30 minutes	18,723	90.5	9,360	94.6	9,363	86.7		7,016	37.5
30+ minutes	1,975	9.5	538	5.4	1,437	13.3		827	41.9
**Education and economic stability**									
AIAN IHS educational attainment: % <high school^a^							<0.0001		
Counties below median	12,009	58.0	6,468	65.3	5,541	51.3		4,448	37.0
Counties above median	8,689	42.0	3,430	34.7	5,259	48.7		3,395	39.1
AIAN IHS income: % < 100% FPL^a^									
Counties below median	9,898	47.8	–	–	–	–		3,437	34.7
Counties above median	10,800	52.2	–	–	–	–		4,406	40.8
AIAN households with incomplete kitchen facilities^b^							<0.0001		
Counties below median	12,730	61.5	8,019	81.0	4,711	43.6		4,705	37.0
Counties above median	7,968	38.5	1,879	19.0	6,089	56.4		3,138	39.4
AIAN households with no vehicle access^b^									
Counties below median	11,648	56.3	9,215	93.1	2,433	22.5	<0.0001	4,255	36.5
Counties above median	9,050	43.7	683	6.9	8,367	77.5		3,588	39.6
**Neighborhood and built environment**									
Low access to a grocery store^c^							<0.0001		
Counties below median	12,964	62.6	6,398	64.6	6,566	60.8		5,210	40.2
Counties above median	7,734	37.4	3,500	35.4	4,234	39.2		2,633	34.0
NCHS 2013 Urban-rural indicator							<0.0001		
Urban	14,494	70.0	7,614	76.9	6,880	63.7		5,527	38.1
Rural	6,204	30.0	2,284	23.1	3,920	36.3		2,316	37.3
**Social and community context**									
Percent AIAN alone or in combination^d^							<0.0001		
Counties below median	7,743	37.4	2,791	28.2	4,952	45.9		3,127	40.4
Counties above median	12,955	62.6	7,107	71.8	5,848	54.1		4,716	36.4

^a^Data source: American Community Survey 2010–2015 5-year estimates.

^b^Data source: US Census Bureau 2000.

^c^Data source: US Department of Agriculture Food Environment Atlas.

^d^Data source: US Census Bureau 2010.

BMI: Body Mass Index (kg/m^2^); AIAN: American Indian/Alaska Native; IHS: Indian Health Service; FPL: Federal Poverty Level; NCHS: National Center for Health Statistics.

Table 3 presents the adjusted odds ratios for obesity among AIAN emerging adults, stratified by county poverty level. Our individual and healthcare results indicated that females generally had higher odds of obesity than males. Individuals who lived at least a 30-minute drive from a primary care facility, which was most often located in a town where additional amenities are located, also have higher odds of obesity relative to those who live closer to a primary care facility. Among our education and economic stability variables, we had some variation across the all-county model to the income stratified models. In the all-county model, individuals who live in counties with lower levels of educational attainment and counties with higher levels of poverty have higher odds of obesity than those who live in counties with more economic resources indexed by education and income (OR Education 1.24, OR Income 1.43). In the model for the lower-poverty counties, individuals living in counties with lower educational attainment have higher odds of obesity; however, this relationship was not found among the higher-poverty model. In the all-county model, we also found that individuals who live in counties with more AIAN households without a vehicle have slightly lower odds of obesity than those who do not. We did not find a statistically significant relationship between health insurance type and odds of obesity. Among our neighborhood and built environment variables, individuals who live in counties with the least access to grocery stores have slightly lower odds of obesity in both the all-county sample model and the higher poverty rate county model; it was not statistically significant in the lower poverty rate model.

**Table 3:** Adjusted odds ratios for obesity for all study population and stratified by income level.

	All sample (n = 20,698)		Lower poverty rate counties (n = 9,898)		Higher poverty rate counties (n = 10,800)	
	OR	95% CI	OR	95% CI	OR	95% CI
**Individual and healthcare**						
Gender						
Male (*reference*)						
Female	1.22	(1.15, 1.30) ***	1.22	(1.11, 1.33) ***	1.21	(1.11, 1.31) ***
Age group						
18–19 years (*reference*)						
20–21 years	1.36	(1.25, 1.47) ***	1.35	(1.20, 1.51) ***	1.37	(1.23, 1.52) ***
22–24 years	1.67	(1.56, 1.80) ***	1.72	(1.55, 1.91) ***	1.63	(1.48, 1.80) ***
Health insurance coverage						
Medicaid						
No Medicaid (reference)						
Had Medicaid	1.05	(0.99, 1.12)	1.23	(1.11, 1.36) ***	0.95	(0.87, 1.03)
Private insurance						
No private insurance (reference)						
Had private insurance	0.99	(0.92, 1.08)	0.99	(0.89, 1.11)	1.00	(0.89, 1.13)
Drive time to primary care						
<30 minutes (reference)						
30+ minutes	1.20	(1.08, 1.34) **	1.27	(1.04, 1.55) *	1.17	(1.03, 1.33) *
**Education and economic stability**						
AIAN HIS county-level educational attainment, ACS 2010–2014: % <high school^a^						
Counties below median (reference)						
Counties above median	1.24	(1.07, 1.43) **	1.29	(1.11, 1.50) **	1.19	(0.93, 1.52)
AIAN IHS income: % < 100% FPL^a^						
Counties below median (reference)						
Counties above median	1.43	(1.22, 1.67) ***	–	–	–	–
County-level AIAN households with incomplete kitchen facilities, Census 2000^b^						
Counties below median (reference)						
Counties above median	0.98	(0.82, 1.18)	1.15	(0.89, 1.49)	1.01	(0.78, 1.31)
County-level AIAN households with no vehicle access, Census 2000^b^						
Counties below median (reference)						
Counties above median	0.82	(0.69, 0.96) *	0.79	(0.62, 1.01)	0.88	(0.68, 1.15)
**Neighborhood and built environment**						
County-level low access to a grocery store, USDA 2010^c^						
Counties below median (reference)						
Counties above median	0.83	(0.72, 0.95) **	0.97	(0.83, 1.13)	0.74	(0.57, 0.96) *
NCHS 2013 Urban-rural indicator						
Urban (reference)						
Rural	0.90	(0.78, 1.06)	0.89	(0.71, 1.11)	0.91	(0.71, 1.17)
**Social and community context**						
Percent AIAN alone or in combination, Census 2010^d^						
Counties below median (reference)						
Counties above median	0.93	(0.80, 1.08)	1.00	(0.85, 1.18)	0.97	(0.74, 1.27)
Fit Statistics for conditional distribution						
-2 log Likelihood (obesity | r. effects)	26860.99					
Pearson Chi-Square	20635.86					
Pearson Chi-Square/ DF	1.00					

*p < 0.05; **p < 0.01; ***p < 0.001.

^a^Data source: American Community Survey 2010–2015 5-year estimates.

^b^Data source: US Census Bureau 2000.

^c^Data source: US Department of Agriculture Food Environment Atlas.

^d^Data source: US Census Bureau 2010.

BMI: Body Mass Index (kg/m^2^); AIAN: American Indian/Alaska Native; IHS: Indian Health Service; FPL: Federal Poverty Level; NCHS: National Center for Health Statistics.

## Discussion

Emerging adulthood, defined as ages 18–24, is a period in which an individual transitions from childhood to adulthood [9]. During this period, individuals become responsible for self-care and develop health related behaviors and habits that will impact future health outcomes [33]. SDOH have been shown to influence underlying health disparities, and these conditions and lack of access to resources are the upstream causes of disease and illness [34]. The emerging adult population of AIAN peoples are a compelling group and understanding the social and environmental contexts in which they live may better inform disease and illness prevention efforts among this group of people. Across the majority of the SDOH domains, we observed a statistically significant relationship between a key variables (e.g. age, poverty, households without vehicle, and access to grocery stores) and obesity among our AIAN emerging adults. It suggests the importance of SDOH in health outcomes and perhaps contributing upstream factor to health status [15,16].

According to the literature, AIAN persons are more likely to be obese relative to their NHW counterparts across the life course; AIAN adolescents are 30% more likely to be obese and 50% of AIAN adults are more likely to have a higher BMI than NHW peers [35]. Unfortunately, experiencing obesity early in life not only confers increased likelihood of physical disability, being obese is also an important risk factor for T2DM, cardiovascular diseases, cancer, and premature mortality [1,36]. Bullock and colleagues found that AIAN childhood obesity prevalence has stabilized from 2006 to 2015 but remains higher than the US overall obesity for children [11]. Our study builds on this important work by examining the prevalence of obesity among emerging adults (ages 18–24) and the association between key SDOH indicators and being obese.

Turning to our neighborhood and built environment variables, individuals who live in counties with the least access to grocery stores have slightly lower odds of obesity in both the all-county sample model and the higher poverty rate county model. The literature indicates that grocery store access does not reliably predict direction on the odds of obesity because the existence of a grocery store does not indicate quality or type of food available to individuals and families [37,38]. There is evidence that limited access to fruits and vegetables is associated with obesity [39]. Notable, AIAN communities are increasing food sovereignty and access to nutrient dense foods within their communities through transforming retail strategies within AIAN owned convenience stores and increasing community gardens with traditional foods [40,41].

Our analysis has limited ability to account for the social and community context because it includes only one measure, percent of AIAN alone or in combination in the county. We did not find a statistically significant relationship between the percent of AIAN alone or in combination in the county and our dependent variable of obesity. Future research should further explore social and community context of AIAN population and the role of stress and the experience of living in these environments and its role on health outcomes.

### Limitations

This study has several limitations. We were limited to one fiscal year of data and thus cannot predict the influence of longer-term SDOH exposure patterns on the overall health of our sample population. Although we have a large sample, the findings are only generalizable to active users of the participating IHS Service Units. Finally, our study uses a person’s BMI to gauge obesity; however, this is a flawed measure that does not adequately account for muscle or bone mass, or body fat distribution [42].

## Conclusion

Our research fills an important gap in understanding the prevalence of obesity among young adults who are American Indian and Alaska Native and the relationship between SDOH county-level indicators and obesity. Our findings suggest the importance of the county environment in shaping body weight. It also reinforces the strong association between lower educational attainment, higher poverty, and obesity.
